# The Importance of Personal Protective Equipment Design and Donning and Doffing Technique in Mitigating Infectious Disease Spread: A Technical Report

**DOI:** 10.7759/cureus.12084

**Published:** 2020-12-14

**Authors:** Robert McCarthy, Bruno Gino, Philip d'Entremont, Ahmad Barari, Tia S Renouf

**Affiliations:** 1 Family Medicine, Memorial University of Newfoundland, St. John's, CAN; 2 Emergency Medicine, Santa Catarina Hospital Intensive Care Unit for COVID-19, Uberlandia, BRA; 3 Pre-Hospital, Sistema Integrado de Atendimento a Trauma e Emergência - Integrated Trauma and Emergency Assistance System, Uberlândia, BRA; 4 Emergency Medicine, Madrecor Hospital, Uberlândia, BRA; 5 General Medicine, University of Limerick, Limerick, IRL; 6 Mechanical Engineering, University of Western Ontario, London, CAN; 7 Mechanical and Manufacturing Engineering, University of Ontario Institute of Technology, Oshawa, CAN; 8 Emergency Medicine, Memorial University of Newfoundland, St. John's, CAN

**Keywords:** personal protective equipment (ppe), novel coronavirus, sars-cov-2, covid 19, simulation medicine, simulation in medical education

## Abstract

During the current coronavirus pandemic, significant emphasis has been placed on the importance of mitigating nosocomial spread of coronavirus disease 2019 (COVID-19). One important consideration involves the appropriate use of effective personal protective equipment (PPE), which may reduce a healthcare provider's likelihood of becoming infected while simultaneously minimizing exposure to other patients that they care for. This may reduce demands placed on the healthcare system and help to preserve the workforce. First, the importance of PPE design cannot be underestimated, as the manufacturing process must strive to maximize protection of the user while ensuring adequate comfort. Second, it has been demonstrated that inadequate education and training can significantly impact compliance with PPE recommendations. Technique regarding donning and doffing of PPE is crucial to the protection of those who don it. The purpose of this technical report is two-fold: first, to describe some important considerations in the manufacturing and design process of face shields to maximize protection for healthcare providers, and second, to describe a simulation scenario that may be used to train healthcare workers in the appropriate donning and doffing of PPE.

## Introduction

Amid the current novel coronavirus pandemic, several recommendations have been communicated to healthcare providers to mitigate spread of infection to patients and among essential healthcare workers. The main mechanism of transmission for coronavirus disease 2019 (COVID-19) is via person-to-person contact (within six feet) by respiratory droplets [1]. Transmission via fomites [1] and aerosols [2] has also been postulated. Significant concerns have been raised about aerosol-based transmission, as several frequently performed procedures, such as provision of high flow oxygen and endotracheal intubation [3], place healthcare providers at particularly high risk of infection. Although individuals are most likely to spread the virus when symptomatic, data suggesting asymptomatic shedding has also been documented. The huge propensity of COVID-19 to spread, and the potentially dire outcomes of such spread within institutions has resulted in several rapid changes to healthcare delivery around the world. Among these, an added emphasis on appropriate use of personal protective equipment (PPE) has been very prevalent [4-6]. At this juncture, protocols for PPE use vary greatly among institutions. Although each may be correct, it is important to have a standardized approach that should be followed by all providers at a particular site, mitigating potential for confusion and contamination in the workplace.

PPE protects healthcare workers from virulent pathogens by preventing exposure to bodily fluids and respiratory droplets [7,8]. The appropriate use of PPE is one of the most effective strategies for protecting both patients and healthcare providers from transmissible pathogens. This strategy becomes especially important when no effective treatment or prophylaxis has been developed for an illness, as is currently the case for COVID-19. When healthcare providers are caring for patients with confirmed or suspected COVID-19, they must follow rigid protocols that necessitate the use of appropriate PPE [8]. The Centers for Disease Control and Prevention (CDC) have released guidelines on the recommended PPE to be worn in various circumstances [4]. In most cases, healthcare providers protect themselves by using a waterproof gown, gloves, a surgical mask, hair protection and a face shield in conjunction with good hand hygiene to minimize mucous membrane exposure to airborne particles [8,9]. Additionally, when providing care to a patient that may involve aerosol-generating procedures, such as endotracheal intubation, appropriately-fitted respirators must be worn [9].

The manufacturing process for PPE significantly impacts its effectiveness in preventing disease transmission. The design of PPE must account for transmission parameters of pathogens along with the physical properties of materials and the environments in which they will be used. Additionally, effectiveness must be balanced with comfort, as the providers donning such equipment are working in stressful environments where unneeded distractions should be minimized. As an example, the utility of face shields has been emphasized during the COVID-19 pandemic [10]. The use of face shields is mandated when healthcare professionals are in close proximity with patients during aerosol-generating procedures to mitigate potential for inoculation onto mucous membranes of the eyes, nose and mouth [11]. In order for a face shield to be effective, it must limit exposure to aerosols and other bodily fluids while also being resistant to fogging. The shield must not adversely impact the vision of healthcare providers, and should be comfortably worn for extended periods of time, even in high-acuity situations. A variety of materials may be used for face shield design, with each having implications with respect to the use of the face shield. As an example, shields with sponges in contact with the forehead are only to be employed as single-use shields, as the spongy materials are not amenable to complete sterility [10].

Aside from factors related to PPE design, meticulous donning and doffing of PPE is a vital step in reducing contamination of healthcare workers caring for patients with transmissible infectious diseases. This is critical to mitigating spread and maintaining the healthcare workforce [12]. Inadequate education and training pertaining to the appropriate use of PPE can negatively impact compliance with recommendations for PPE use [7]. Therefore, educating providers about PPE during this global pandemic may prove effective in reducing the spread of COVID-19.

Additional strategies to minimize spread

Environmental control is also vital in minimizing the spread of this novel coronavirus. When patients are seen in the Emergency Department (ED), it is advised that they be triaged quickly and placed in a private room, isolating them from other patients [1]. In the event that a patient is critically ill, they should ideally remain in an airborne isolation room (or negative pressure room), particularly if aerosol-generating procedures will be required. Traffic in and out of the room must be minimized.

Several other strategies to mitigate spread among healthcare workers are being implemented worldwide. Among these, important personal protective measures include limiting personnel in a room when caring for critically ill patients, clearly communicating a patient’s COVID-19 screening status to any providers in the circle of care and prioritizing oxygenation and ventilation strategies with lower aerosolization risk [5]. If intubation is deemed necessary, cuffed endotracheal tubes and high-efficiency particulate air (HEPA) filters should be utilized at all times. To minimize the number of pass attempts, the healthcare provider with the most experience managing an airway should be involved. Fibreoptic intubation or video laryngoscopy are suggested over direct laryngoscopy so that the spread of respiratory droplets can be further reduced if a patient coughs. In recent months, added emphasis has been placed on the use of simple surgical masks for all providers and patients during clinical encounters, and this has been shown to be quite effective in reducing transmission as well [12]. Although adherence to these strategies does not eliminate the inherent risks involved in providing care to affected patients, they can help maximize the safety of essential healthcare workers.

Role of simulation-based medical education (SBME)

Simulation-based medical education (SBME) has proven to be a very effective strategy in teaching clinical and team-based communication skills [13]. In the current clinical climate, appropriate donning and doffing of PPE is a crucial step in direct patient care, and simulation-based training is one strategy that may help to ensure these skills are acquired by healthcare providers. Evidence from a prior pandemic suggests that simulation training can help to develop competence in PPE utilization among healthcare providers [14].

The major learning objectives of this technical report are two-fold. First, we aim to introduce some important considerations in the manufacturing of PPE (focusing on face shields) and how research in this area may be facilitated through virtual simulation. Second, we describe a simulation scenario that focuses on developing the necessary knowledge and skills for healthcare providers to take appropriate protective actions when treating patients who are potentially infected with COVID-19. The learning objectives related to the simulation scenario, specifically, are as follows:

1) Demonstrate how to appropriately don and doff PPE in the context of caring for patients with highly transmissible diseases, such as COVID-19.

2) Develop an initial approach to managing patients suspected to have COVID-19 in the ED setting, mainly focusing on patient and provider safety, along with initial stabilization measures.

3) Demonstrate an approach to managing additional patients who are receiving care in the ED when a patient presents with a highly transmissible disease, such as COVID-19, focusing specifically on reduced transmission to other patients.

## Technical report

Design effectiveness

As mentioned previously, specific design can impact the effectiveness of PPE in several ways. When considering face shield models as an example, there are multiple factors to consider when trying to optimize the utility of this protective equipment. First, the fit must effectively limit exposure to aerosols and be resistant to fogging [11]. It must also allow for adequate vision for users, and be comfortable enough to be worn for extended periods of time and in high-stress situations. The use of different materials may mandate that shields are disposable, as some components, such as the sponge commonly used to facilitate comfort along the user's forehead, is not amenable to adequate sterilization [10]. Given all of these considerations, evaluating the design of PPE must consider user feedback and scientifically derived data as modifications are made.

**Figure 1:**
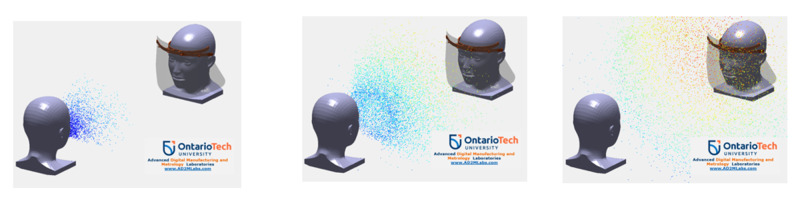
Computational Dynamics Modelling of COVID-19 Transmission From Infected Patient to Personal Protective Equipment (PPE) User via Air at 10 ms, 20 ms and 50 ms (Left to Right) Following a Sneezing Event.

A practical way to complete a design effectiveness evaluation (DEE) for PPE is to develop a virtual simulation environment using computer-aided evaluation (CAE) methods. The simulation is developed considering the details of the transmission parameters and the corresponding physical properties. In order to accurately measure and illustrate probability of COVID-19 transmission, using detailed knowledge of airflow patterns and particle distribution, mathematical modelling may be utilized to evaluate how droplets are aerosolized and spread across a variety of physical distances. This can be achieved by creating a 3D computer-generated model of a user’s face, face shield, and the environment and then setting parameters for droplets and air including temperature, density, viscosity, and airflow, among others. Figure 1 presents the examples of CAE simulation for the preliminary analysis on a PPE face shield demonstrating transmission of viral particles following a sneezing event at times of 10, 30, and 50 milliseconds, respectively.

**Figure 2:**
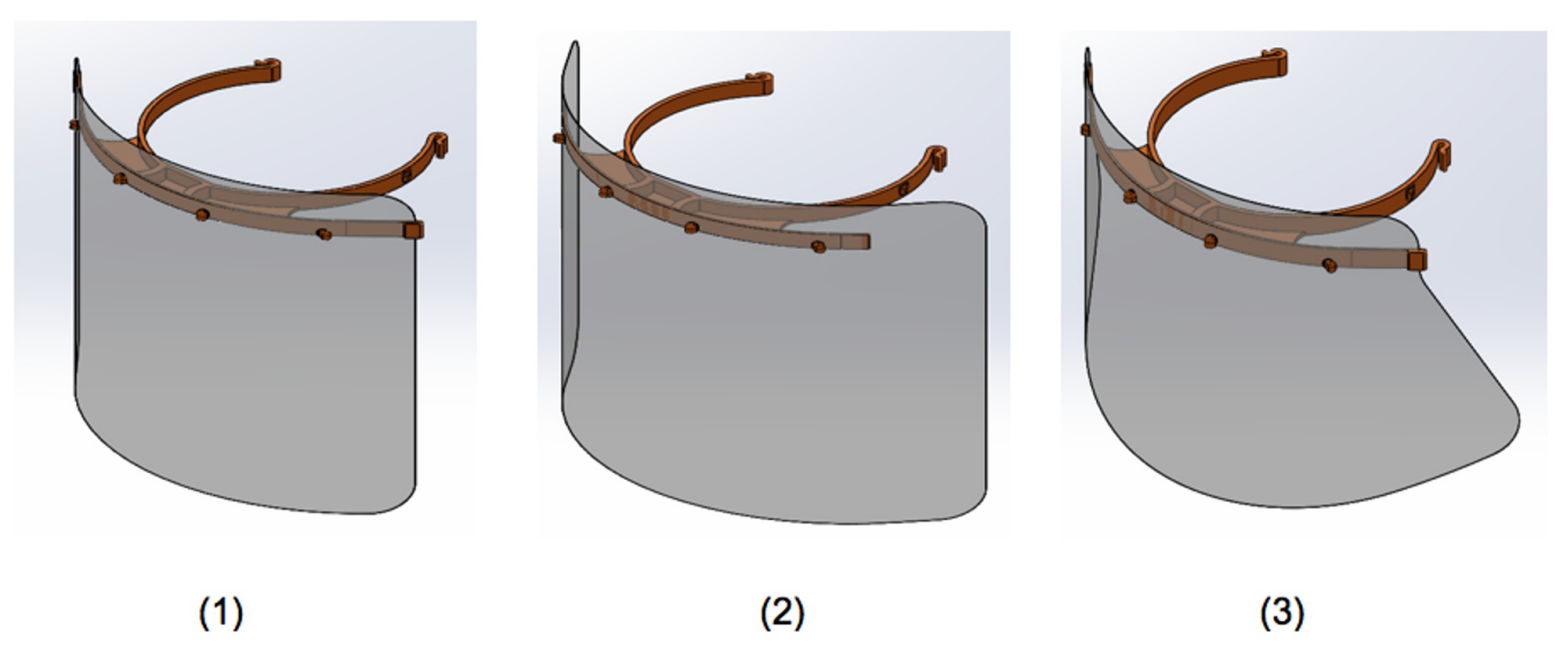
Comparing the Efficiency of Three Designs of Personal Protective Equipment (PPE).

The virtual simulation environment can be used to find the optimum design variables in a Closed Loop between design variation and virtual simulation [15]. As a result, various designs of PPE can be compared to determine the one that is most effective under different scenarios. In an example, three different face shield designs were tested for the same scenario, as outlined in Figure 2. When variables such as the distance from the patient, the room temperature, the density of the air and air flow were held constant, design 2 proved to be most effective in terms of preventing particles from entering the user’s airway. Design 1 was rated at 81.8% effectiveness of design 2, while design 3 received an effectiveness score of 75.8% relative to design 2. Therefore, when considering viral transmission, PPE design must also be considered crucial in optimizing the safety of users who don it.

The effectiveness of PPE must be balanced against several practical aspects pertaining to the context in which PPE is used. Oftentimes, PPE is required in high stress situations in which providers are caring for sick patients [11]. As such, the donning process for PPE must be easy and efficient, so as to maximize the time spent providing direct patient care. Additionally, PPE design must also consider a user’s comfort, as providers must be able to perform procedures and think through difficult patient scenarios without being distracted by uncomfortable or intrusive equipment. Therefore, striking a balance between effective protection and user comfort should be prioritized by institutions when choosing the PPE to provide to their employees. Although not emphasized throughout our simulation scenario, it is noteworthy that virtual simulation can be used to optimize protection of healthcare providers, specifically when considering the engineering and manufacturing process of PPE.

Simulation scenario

Pre-Scenario

You are the attending emergency room physician working in a rural ED. In the middle of what has been a busy shift, you receive a call from Emergency Medical Services (EMS) stating that they are en route with a 65-year-old male who is acutely short of breath. About 30 minutes ago, his wife contacted EMS as her husband had become very dyspneic. He has not been feeling well for the last three days, with intermittent fever, chills and a severe headache. They returned from a trip to New York City seven days ago. The patient’s current vitals are: blood pressure (BP) 110/70, respiratory rate (RR) 24, temperature 38.4 deg C, heart rate (HR) 120, oxygen saturation (SpO2) 88% on 5L via nasal prongs. They are about 10 minutes out.

Context and Inputs

Learners begin the case after having been briefed on the content of the phone call from EMS. They are expected to begin preparing for the arrival of a 65-year-old male who is acutely short of breath in the context of a febrile illness and recent travel (as described above). The emergency department is currently staffed by one ER physician and two registered nurses. The hospital has access to an off-site respiratory therapist on call. There are currently two other patients in the ER. First, a 78-year-old male who presented with chest pain is present in the resuscitation room awaiting a second troponin result. His initial electrocardiogram (ECG) showed anterior ST-depression and he is on continuous cardiac monitoring. He is vitally stable. The second patient is a 35-year-old female located in the minor procedures room awaiting assessment after cutting her hand at home. She is vitally stable but is distressed and in some pain. The main skills to be assessed throughout the simulation scenario include demonstrating the appropriate technique for donning and doffing PPE. Additional skills include demonstrating an approach to the initial management of a patient with respiratory distress in the context of the COVID-19 pandemic, being cognizant of patient and provider safety.

This simulation scenario is appropriate for three learners - one can take on the role of the ER physician and two others can play the roles of nurse. Two paramedics and the respiratory therapist may be played by confederates. If less than three learners are available, the role of nurse may also be assumed by confederates. This simulation scenario is appropriate for ER physicians as well as emergency medicine residents. Table 1 outlines the equipment used for running the simulation scenario. Of note, the only equipment needed by participants is the appropriate PPE, as participants are expected to demonstrate appropriate donning and doffing technique. All additional equipment may enhance the realism of the simulation, but should not need to be used during the scenario.

**Table 1:** Available Equipment for the Simulation Scenario. ECG: Electrocardiogram

Personal Protective Equipment:	
Gloves (1 pair for each participant)	Face shield (1 for each)
Waterproof Gown (1 for each participant)	N95 mask (1 for each)
Closed toe shoes	
Other:	
Hand sanitizer	Bag-Mask Ventilator
Nasal Cannula	Non-Rebreather Facemask
Surgical Masks	Crash Cart
Stretcher	ECG Leads & Pacer Pads
Blood Pressure Cuff	Monitor or iPad to simulate monitor with vitals

We recommend that one emergency medicine physician and one emergency room nurse be available to assess participants. The emergency medicine physician should be able to provide feedback regarding management of the emergency department, including preparation prior to a patient’s arrival. The nurse should be proficient in donning and doffing of PPE and be able to provide feedback to participants regarding their technique. It is recommended that instructors run through the scenario ahead of time in order to identify limitations or technical issues with the running of the simulation.

Process

This simulation scenario may involve the use of one standardized patient (SP), playing the role of the male patient who has been transported to the ED via ambulance. The healthcare team will include two paramedics who may be played by confederates, along with an ER physician and two ER nurses, whose roles will be assumed by participants. If less than three participants are involved, nursing roles may also be assumed by confederates.

Prior to starting the simulation scenario, participants will be briefed on the fictional contract, which requires acknowledging that although the simulated case is fictional, they are to treat it as if it is ‘real’, acting as they would in clinical practice so as to make the simulation worthwhile. Learners should be advised that the simulation is purely formative, and that constructive feedback will be provided to each learner once the scenario is over. Instructors and any confederates should be introduced to learners at this time, highlighting each person’s role within the team. Learners should be briefed on how to order investigations and obtain results. Any limitations associated with the simulation should be discussed. For example, this simulation scenario will not focus on intubation of this patient population, and therefore learners may just verbalize if they feel this intervention may be required. The scenario will end when participants have appropriately isolated and ‘stabilized’ the patient.

Table 2 is a detailed, stepwise scenario template. This should be provided to instructors approximately two weeks prior to the running of the simulation scenario. This will allow the team to adequately prepare confederates and SP’s for their respective roles during the simulation. We suggest performing a practice run to identify any other limitations or technical issues with running the simulation. An important point to consider is that protocols around the appropriate use of PPE are subject to changes based on emerging data, especially in the context of a novel pathogen such as COVID-19. Additionally, PPE protocols can vary slightly from one institution to another. Therefore, it is crucial for instructors to verify the accuracy of this template in the context of the institution in which they are teaching and to educate participants about the evolving nature of the information provided.

**Table 2:** Simulation Scenario Design With Modifiers. EMS: Emergency Medical Services; RT: Respiratory Therapist; IM: Internal Medicine; PPE: Personal Protective Equipment; ED: Emergency Department; O2: Oxygen; NP: Nasal Prongs; VS: Vital Signs; HR: Heart Rate; BP: Blood Pressure; RR: Respiratory Rate; SpO2: Oxygen Saturation; T: Temperature; GCS: Glasgow Coma Scale; CBC: Complete Blood Count; LBC: Electrolytes, Blood Urea Nitrogen, Creatinine; CXR: Chest X-Ray; VBG: Venous Blood Gas

STATE	STATUS	LEARNER ACTIONS	OPERATOR NOTES	LEARNING OBJECTIVE
1.Preparation (following phone call from EMS)		Gather Team & Assign roles		Initial approach, team safety (2)
		Provide overview of case to team	*Modifier: If not mentioned in the briefing, nurse should ask if patient is at risk for COVID-19.	Initial approach, team safety (2)
		Call for additional help (RT, IM)		Initial approach (2)
		Ensure appropriate PPE available	*Modifier: If not mentioned, nurse to ask if appropriate PPE readily available	Initial approach, team safety (2)
		Ensure crash cart available, with necessary supplies for intubation	*Modifier: Nurse to prompt if not asked for	Initial approach (2)
		Move patient awaiting troponin to separate room	*Modifier: If no mention of moving patient, nurse to ask where he should go	Management of additional patients in ED (3)
		Provide surgical masks to both patients already present in ER	*Modifier: If not addressed by team, nurse to prompt participants by asking whether these patients require masks.	Patient safety (2), management of additional patients in ED (3)
	Both patients vitally stable	Quick assessment of both patients in ED		Management of additional patients in ED (3)
		Ensures team has appropriate PPE available (including appropriate N95 mask)		Initial approach, team safety (2)
		Instructs team to not enter room until all PPE has been donned	Trigger: When preparations made, → 2	Team safety (2)
2.Donning PPE/Patient arrival		Each participant follows orderly, stepwise approach		Donning & Doffing PPE (1)
		Washes hands		Donning & Doffing (1)
		Puts on appropriate gown and ties all ties		Donning & doffing (1)
		Selects appropriate N95 Mask and puts it on appropriately		Donning & doffing (1)
	Patient arrives. On 4L O2 by NP. VS HR 120 BP 110/60 RR 24 SpO2 87% T 38.5 C GCS 14 Appears confused and anxious, unable to speak in full sentences, increased work of breathing with accessory muscle use	Patient to be brought to resuscitation room for further assessment	Modifier: One nurse goes to enter room with patient without all PPE donned. Physician should stop her and re-iterate the importance of appropriate PPE	Donning & doffing (1), Initial approach (2)
		Applies face shield		Donning & doffing(1)
		Sanitizes hands again		Donning & doffing (1)
		Dons appropriately- sized gloves		Donning & doffing (1)
		Ready to enter room	Modifier: All bodies go to enter room. Physician suggests that one nurse remain outside to minimize contact for now → 3	Donning and doffing (1), Team safety (2)
3.Patient Management		Set up continuous monitoring		Initial approach (2)
		High flow O2 with surgical mask placed over patient’s face		Initial approach (2)
	VS improving BP 115/65 HR 110 RR 20 SpO2 88% GCS 15 Work of breathing decreased, no longer seems confused. Less anxious.	Order Investigations: CBC, LBC Blood Cultures Portable CXR VBG, lactate		Initial approach (2)
		Patient will not require intubation at this time		Initial approach (2)
4.Doffing PPE appropriately		Breaks ties on gown		Donning & doffing (1)
		Removes gloves without contaminating hands (may use “Bird Beak” or “Glove in Glove” technique to do so)		Donning & doffing (1)
		Removes gown using rolling technique		Donning & doffing (1)
		Washes hands		Donning & doffing (1)
		Leaves room		Donning & doffing (1)
		Removes faceshield and mask		Donning & doffing (1)
		Washes hands	End of Scenario	Donning & doffing (1)

Following the completion of the simulation, all participants will be involved in a debriefing session that encourages reflection and promotes formative feedback. A short didactic session will be offered to cover the learning objectives of the scenario, as well as highlight the key differences in resuscitative protocols when caring for a COVID-19 positive patient.

Product/Outcomes

A checklist for formative assessment of the scenario is included below, with each step mapped to the appropriate learning objective(s) that are listed in the introduction (Table 3).

**Table 3:** Checklist for Formative Assessment of Learners. PPE: Personal Protective Equipment; ED: Emergency Department; ECG: Electrocardiogram; BP: Blood Pressure; O2 sat: Oxygen Saturation; IV: Intravenous

Learning Objective #1: Demonstrate how to appropriately don and doff PPE in the context of caring for patients with highly transmissible diseases, such as COVID-19.		
	Yes	No
Addresses need to obtain appropriate PPE		
Dons PPE Appropriately:		
Washes hands		
Puts on gown and ties		
Selects appropriately-sized mask and dons correctly		
Dons faceshield		
Re-washes/sanitizes hands		
Dons appropriately-sized gloves		
Doffs PPE Appropriately:		
Removes gloves without touching exterior surface using “Glove-in-Glove” or “Bird Beak” technique, as discussed in Table 5 below.		
Sanitizes hands		
Removes gown using appropriate “rolling” technique		
Sanitizes hands		
Leaves room prior to removing face shield and mask		
Removes face shield without touching external surface		
Removes mask by headpieces (without touching exterior surface)		
Re-sanitizes hands		
Learning Objective #2: Develop an initial approach to managing patients suspected to have COVID-19 in the ED setting, mainly focusing on patient and provider safety along with initial stabilization measures.		
Gathers team		
Provides information regarding patient en route		
Assigns roles appropriately		
Explains importance of minimizing number of people in the room		
Calls for additional help		
Ensures crash cart and airway kit is available if needed		
On arrival of patient:		
Continuous ECG monitoring		
Intermittent BP monitoring		
Continuous O2 Sat		
Obtains IV access		
Advises application of surgical mask to patient on arrival		
Delivers O2 via nasal cannula (may apply surgical mask over NC, as recommended in some resuscitation guidelines)		
Interrupts staff from treating patient until all PPE has been donned		
Orders appropriate initial investigations		
Mentions potential need to intubate		
Re-assesses patient frequently		
Learning Objective #3: Demonstrate an approach to managing additional patients who are receiving care in the ED when a patient presents with a highly transmissible disease, such as COVID-19, focusing specifically on reduced transmission to other patients		
Moves patient from resuscitation bay to clear room for potentially infectious patient		
Provides additional patients in ED with surgical masks		
Quickly reassesses stability of additional patients prior to arrival of patient en route		
Calls for additional help for other patients if required		
Global Performance		
Effective Communication with team members		
Appropriate attention to safety of colleagues		
Appropriate attention to patient safety		
Appropriate attention to personal safety		

Debriefing

Following the completion of the scenario, all learners will be provided with an opportunity to debrief, highlighting whether their experience was positive or negative. They will be given time to ask questions pertaining to the simulation and should also be encouraged to provide feedback regarding the simulation design and execution. The debriefing should be facilitated by the two instructors and should follow an advocacy-inquiry type of design [16]. Facilitators should provide constructive feedback to each learner and subsequently allow learners to reflect on their experience while participating in the scenario.

**Figure 3:**
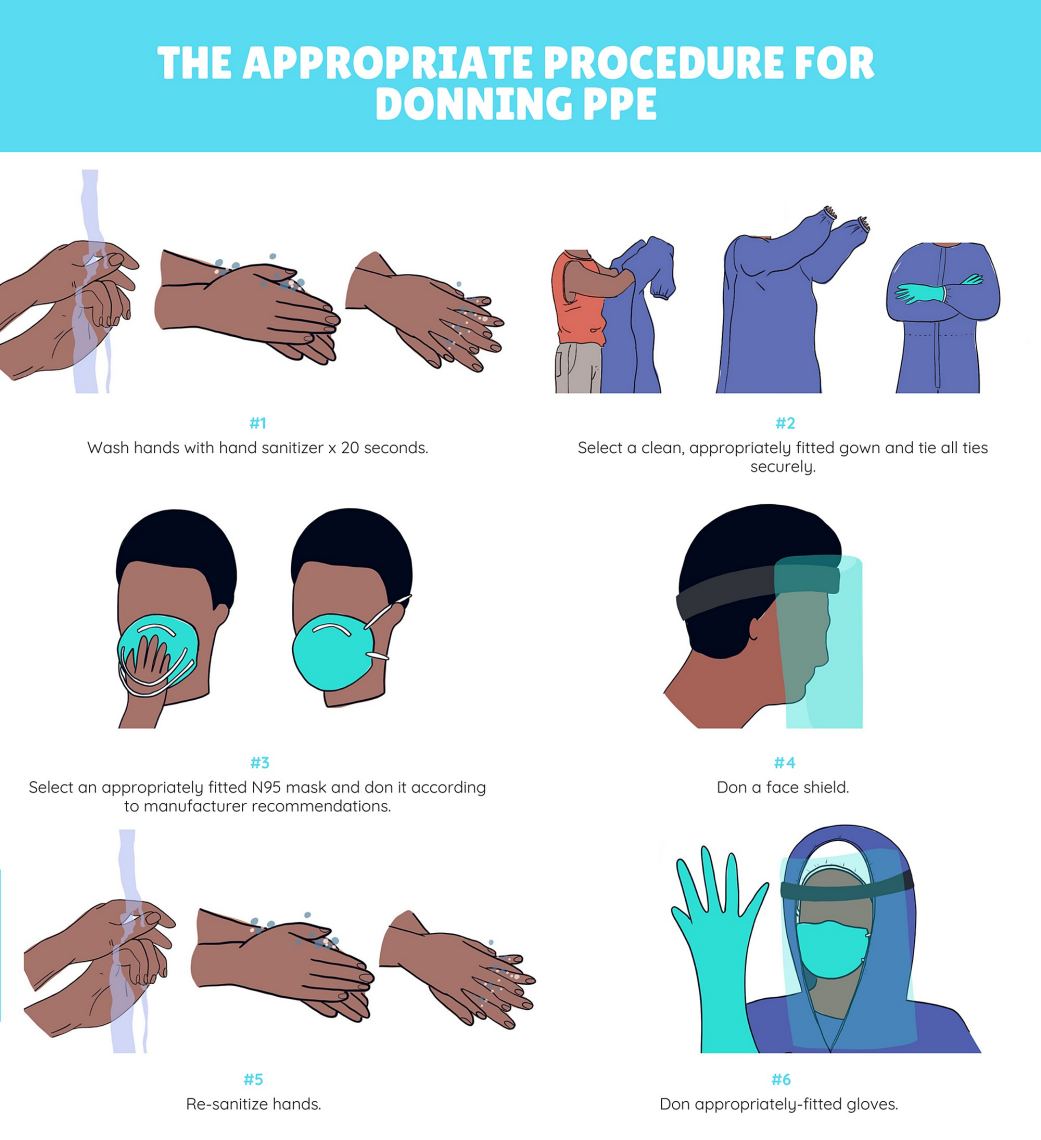
Diagram Demonstrating Appropriate Donning of Personal Protective Equipment. ^Artwork Acknowledgement: Mirna Nogueira de Alencar, Fashion Design, Pontifical Catholic University of Parana, Brazil^

**Figure 4:**
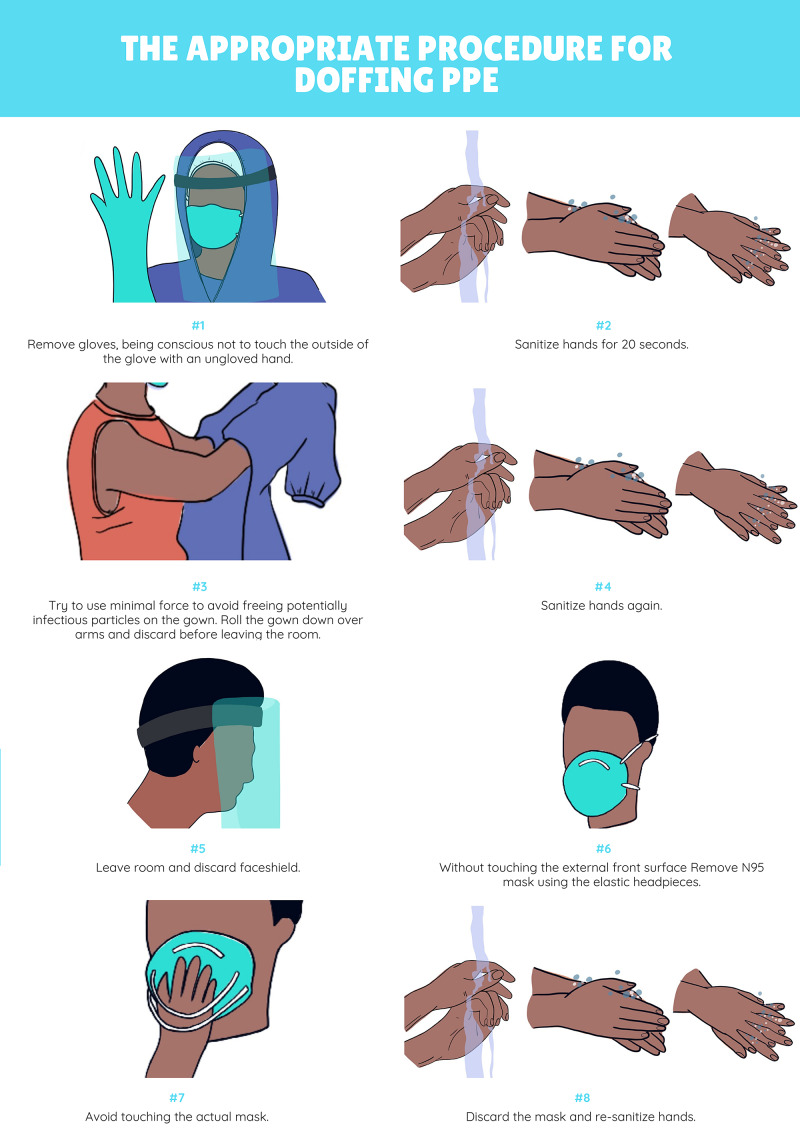
Diagram Demonstrating Appropriate Doffing of Personal Protective Equipment. ^Artwork Acknowledgement: Mirna Nogueira de Alencar, Fashion Design, Pontifical Catholic University of Parana, Brazil^

Following the debrief, approximately 30 minutes of didactic instruction is included to provide additional teaching related to the learning objectives covered in the case. The inclusion of a didactic session immediately following a simulation has been shown to consolidate key clinical information and to highlight knowledge gaps for future learning [13]. The main teaching points to be covered in the didactic session are included in Table 4. Figure 3 and Figure 4 are diagrams that may be used to illustrate the appropriate technique for donning and doffing of PPE to participants. See Table 5 for glove doffing techniques.

**Table 4:** Teaching Points for the Didactic Session. PPE: Personal Protective Equipment; ED: Emergency Department; O2: Oxygen; NRB: Non-Rebreather; CPAP: Continuous Positive Airway Pressure; BiPAP: Bilevel Positive Airway Pressure; NP: Nasopharyngeal

Learning Objective	Teaching Points
Demonstrate how to appropriately don and doff PPE in the context of caring for patients with highly transmissible diseases, such as COVID-19.	The appropriate procedure for donning PPE is as follows [4,6]: Wash hands with hand sanitizer x 20 seconds Select a clean, appropriately fitted gown and tie all ties securely. Select an appropriately fitted N95 mask and don it according to manufacturer recommendations Don a face shield Re-sanitize hands Don appropriately-fitted gloves In order to doff PPE correctly, the following procedure should be followed: Remove gloves, being conscious not to touch the outside of the glove with an ungloved hand (See Table 5 for doffing techniques) Sanitize hands for 20 seconds Break ties on the gown. Try to use minimal force to avoid freeing potentially infectious particles on the gown. Roll the gown down over arms and discard before leaving the room. Sanitize hands again Leave room Discard faceshield without touching the external front surface Remove N95 mask using the elastic headpieces. Avoid touching the actual mask. Discard and re-sanitize hands. See Figure 4 for a schematic representation of the donning and doffing process that may be used as a visual aid during didactic teaching.
Develop an initial approach to managing patients suspected to have COVID-19 in the ED setting, mainly focusing on patient and provider safety along with initial stabilization measures.	Prior to the arrival of a patient who is potentially infected with a highly transmissible disease, such as COVID-19, it is very important for the emergency room physician to update their team to ensure everyone is adequately prepared. The team must ensure that appropriate PPE, including a clean, fluid-resistant gown, gloves, a face shield and an appropriately fitting N95 mask is available for anyone who will provide direct patient care [5]. All PPE must be appropriately donned before attempting to treat the patient. Team safety is paramount in order to preserve the workforce and maximize safety. Any other patients in the ED must be moved to a separate location from the room where the potentially infectious patient will be treated. Ideally, high-risk patients should be treated in a negative-pressure room. The team should minimize the number of people who will provide direct patient care in order to mitigate the potential for spread. The person who is most experienced with airway management should be prepared to intubate if required, in order to minimize the number of passes needed during this aerosol-generating procedure. In the event that a patient requires respiratory support, attempts to oxygenate and ventilate the patient should move quickly from oxygen via nasal cannula to endotracheal intubation. If possible, direct laryngoscopy should be avoided with use of video-assisted laryngoscopy advocated to minimize proximity of provider to patient.
Demonstrate an approach to managing additional patients who are receiving care in the ED when a patient presents with a highly transmissible disease, such as COVID-19, focusing specifically on reduced transmission to other patients	This learning objective mainly focuses on the protection of other patients who may be in close proximity with the potentially infectious patient during their time in the ED. In order to minimize contact, the infectious patient should be brought to an isolation room under negative pressure (if available) or at least be treated in a unit that contains no other patients. In our particular case, every effort should be made to protect the other patients by moving them to separate areas of the ED and providing them with surgical masks. Since these patients will not be in close proximity with the affected patient, they do not require N95 masks or other PPE if they are moved in a timely fashion.
Additional Didactic content may include the differences in PPE requirements for Aerosol and Non-Aerosol generating procedures.	1) Aerosol-Generating Medical Procedures [3]: The following procedures are considered high-risk and, when performed, require all healthcare providers in close proximity to don high-level PPE, including use of an N95 mask: O2 above 5L/min by NP O2 above 15L/min by Venturi or Non-Rebreather (NRB) mask Non-Invasive Ventilation (CPAP/BiPAP) Provision of nebulized medication Intubation and extubation Tracheotomy or tracheostomy Bronchoscopy Any procedures that induce coughing or collection of sputum Lower risk procedures require only use of a simple surgical mask in place of an N95 respirator. These include: Collection of NP/Throat Swab Chest tube removal/insertion (unless in setting of urgent pneumothorax) Oral hygiene Any procedure with regional anesthesia Chest physiotherapy (other than breath stacking) O2 delivered below 6L/min by nasal prongs or 15L/min by Venturi or NRB Intranasal medication, such as naloxone

**Table 5:** Two Techniques for Appropriate Removal of Contaminated Gloves [17].

Glove-in-Glove Technique	Bird Beak Technique
Use index finger and thumb to grab outside part of glove near wrist without touching skin	Using the index finger and thumb of a gloved hand, pinch the top of the other gloved hand near the wrist, pulling the glove away from the skin.
Pull glove away from exposed skin, being sure not to touch any exposed skin	Using the middle finger of the pinching hand, scoop up the edge of the glove, pulling it further from the wrist.
Peel glove away from hand, turning it inside out in the process of pulling it towards the fingers	Using the index finger, middle finger and thumb, pull the glove inside-out over the fingers to form a “bird beak”.
Continue to pull the glove away and do not let go of it as it leaves the hand	Use the bird beak to grasp the top of the other glove near the wrist
Hold the removed glove in the gloved hand used to remove it	Using the beaked hand to pull the second glove off, peeling it away from the hand and causing it to go completely inside-out. Do not let go of the second glove with the beaked hand.
Slip the index finger of the exposed hand underneath the remaining glove near the wrist, being sure not to touch any external surface of the glove or gown	Using the index finger of the newly exposed hand, peel away the glove from the beaked hand.
Pull the second glove inside out while peeling it off the hand while continuing to grasp the glove that was first removed. As the glove is rolled completely inside out, the previously removed glove will be contained within it. These may then be discarded and handwashing should commence immediately.	When both gloves are completely removed, dispose of them appropriately and proceed to proper handwashing.

This scenario is designed to demonstrate appropriate precautions for healthcare providers while caring for patients with potentially transmissible infectious diseases. It has been tailored to highlight many relevant concerns in the setting of the current novel coronavirus pandemic.

The appropriate use of PPE can play a significant role in mitigating the nosocomial spread of infectious diseases [12]. This simulation scenario emphasizes the importance of PPE, both for protection of healthcare providers, as well as their patients. This step should be the top priority for healthcare professionals when they encounter a patient who has potentially been infected by the coronavirus, with complete donning of PPE required before any close patient contact ensues [5].

Other important considerations that are covered in this scenario are very relevant in the context of the current coronavirus pandemic. Given the transmissibility of this pathogen, extra weight has been placed on the protection of the healthcare team involved in caring for potentially affected individuals. First, recommendations from multiple organizations suggest limiting the number of providers involved in patient care to the fewest needed to meet the patient’s care needs. When patients require intubation, the procedure should be delegated to the most experienced provider to minimize the number of potential passes [5]. Video laryngoscopy should be used to reduce the proximity between patient and provider during the procedure when available. Pre-oxygenation of patients should be achieved via nasal cannula or a non-rebreather facemask covered with a surgical mask. Finally, when considering the need for cardiopulmonary resuscitation (CPR), the risk of exposure to the team should be weighed against the chances of a successful outcome. Although outcomes of CPR in the context of COVID-19 are not yet known, guidelines recommend considering age, co-morbidities and severity of illness when determining whether or not to proceed.

## Discussion

There were two major objectives to this technical report: first, to describe important considerations in the manufacturing process of PPE, linking it with virtual simulation-based methods, and second, to describe a simulation scenario that can be used to train healthcare providers in the appropriate donning and doffing of PPE. 

The overarching theme of this report aims to teach important principles related to the protection of healthcare providers when treating patients suspected of having highly transmissible infectious diseases. Throughout the report, we highlight several important considerations in the design process, which must balance provider safety with comfort. Given the evolving knowledge base regarding COVID-19, studying the factors that impact provider comfort and safety are worthwhile. User feedback [11] and scientific evidence gleaned through virtual simulation can help to inform future changes to further improve provider comfort and safety.

Our simulation scenario was designed to teach healthcare providers the appropriate way to don and doff PPE. Although the case emphasizes donning and doffing in the context of COVID-19, the scenario may be adapted for other clinical situations. One important consideration is that PPE donning and doffing protocols can vary slightly from one institution to another. As such, facilities offering this simulation opportunity within their workplace are encouraged to review the PPE protocol and to make adjustments to fit with local regulations. 

Overall, simulation-based education is effective in teaching clinical and team-based communication skills [13]. The meticulous donning and doffing of appropriately designed personal protective equipment in accordance with an institution's protocol is a vital step in mitigating the spread of COVID-19 and, in turn, maintaining the healthcare workforce [9]. It has been demonstrated that inadequate education and training can negatively impact compliance with PPE protocols [7]. Therefore, the above simulation may help to promote compliance among healthcare professionals who complete it. 

## Conclusions

In the context of the coronavirus pandemic, it is essential for healthcare providers to protect themselves from potential exposure as much as possible. This protection begins by having access to effective PPE on a regular basis. Optimizing PPE design is critical, striking an appropriate balance between adequate protection, comfort and ease of use. Regardless of the PPE being distributed, becoming proficient in donning and doffing this equipment is paramount. Simulation-based training, as proposed in this article, may allow healthcare providers to improve their knowledge and technique regarding appropriate PPE use. We surmise that this may contribute to reduced rates of nosocomial spread of COVID-19.
